# Patellar tracking and patellar tilt in kinematic versus mechanical knees

**DOI:** 10.1002/jeo2.70848

**Published:** 2026-07-18

**Authors:** Meredith Benson, Chase Gornbein, Mitchell Pfennig, Steven Denyer, Nicholas Brown

**Affiliations:** ^1^ Loyola University Chicago Stritch School of Medicine Maywood Illinois USA; ^2^ Department of Orthopaedic Surgery and Rehabilitation Loyola University Medical Center Maywood Illinois USA

**Keywords:** kinematic alignment, patellar tilt, patellar tracking, total knee arthroplasty

## Abstract

**Purpose:**

This study aims to compare patellar tracking and tilt following kinematic and mechanical total knee arthroplasty (TKA) via comparison of the incidence of lateral release and the patellar tilt angle on the postoperative sunrise X‐ray view.

**Methods:**

A retrospective study was conducted identifying 252 patients who underwent TKA. Radiographic measurements were collected prior to and following TKA, including the mechanical distal femoral angle, proximal tibial angle, lateral joint line release, patellar tilt and patellar displacement prior to and following operation.

**Results:**

Of the 222 patients analysed, 119 patients underwent kinematic TKA (KA‐TKA) and 103 patients underwent mechanical TKA (MA‐TKA). Mechanically aligned knees were found to have a higher incidence of lateral joint line release (3.9% vs. 0%) (*p* = 0.04) and a greater incidence of patellar retinacular release (4.9% vs. 0.8%), although not significant (*p* = 0.10). There was no difference between kinematic and mechanical TKA regarding change in patellar tilt or patellar displacement prior to and following TKA.

**Conclusion:**

Patellar tracking was found to be comparable between mechanical and kinematic TKA, with no significant difference in incidence of patellar release, patellar tilt or patellar displacement. Mechanically aligned TKA required a non‐statistically significant increased incidence of lateral joint line release. Overall, these results support that MA‐TKA and KA‐TKA both result in satisfactory patellar tracking with possible reduced need for patellar and lateral joint line releases in KA‐TKA.

**Level of Evidence:**

Level III, clinical research.

**Abbreviations**
dFA: distal femoral angle
KA: kinematic alignment
KA‐TKA: kinematically aligned total knee arthroplasty
MA: mechanical alignment
MA‐TKA: mechanically aligned total knee arthroplasty
pTA: proximal tibial angle
TKA: total knee arthroplasty

## INTRODUCTION

The alignment of total knee components is a crucial step in a knee replacement surgery, as malalignment of components is associated with aseptic loosening, earlier polyethylene wear and increased pain [4, 19]. Traditionally, knee arthroplasty is performed in what is known as the mechanical alignment, indicating that the joint line is replaced perpendicular to the mechanical axis. While this has served as a trusted standard, the native knee is not found in a completely neutral position, and in the previous 15 years, there has been a recent shift towards kinematically aligned total knee arthroplasty (KA‐TKA), where the components are placed with the goal of restoring pre‐arthritic alignment and rotation [28]. This offers benefits including ligament tension and balance maintenance, theoretically placing less stress on ligaments when they are in a more natural position [30].

The kinematic knee has gained interest as it is theorised to have improved patient satisfaction due to a more ‘natural’ feeling. A few studies have shown the use of this alignment can provide better pain relief, improved function and increased range of motion, as well as a more ‘normal’ feeling of the knee [8, 25]. Not all randomised control trials were able to show a positive relationship between kinematic alignment and improved outcomes [32]. However, a recent meta‐analysis by Courtney and Lee showed no difference in complication rate between the two alignment methods did find improvements in postoperative Knee Society Score in the kinematically aligned group [9]. Similarly, Howell et al. and others have shown that kinematically placed knee arthroplasty did not negatively impact 10‐year implant survival, yearly revision rate, or level of function [15]. A further follow‐up of the same cohort at 16 years showed a 93% implant survivorship with a lower reoperation rate than mechanical TKA [13]. Finally, KA‐TKA has been found to offer advantages in the postoperative period including improved postoperative pain, shorter length of hospitalisation and decreased opioid requirements [10, 20].

Patellar maltracking is a relatively common complication following knee arthroplasty, with patellofemoral instability reported in up to 20% of patients following TKA [3, 7, 11]. Maltracking of the patella is traditionally thought to be the result of rotational malalignment of either the tibial or femoral component [5, 24]. Multiple studies specifically emphasise that increased internal rotation of the femoral or tibial component may contribute to altered patellofemoral kinematics and post‐operative anterior knee pain [6, 21]. If there is concern for lateral patellar tilt, an intraoperative lateral retinacular tissue release can be performed to ensure a more midline tracking of the patella, with minimal complications [18].

In a typical mechanical knee, the femoral component is often externally rotated to assist with tracking. Conversely, in performing a kinematic knee arthroplasty, both the femoral and tibial components are rotated to match native anatomy, often more internally than in mechanical knees. In addition to internal rotation, the knee is frequently cut with a greater degree of valgus alignment to match native knee anatomy, resulting in an increased quadriceps force vector and more medially oriented trochlear groove relative to a mechanical knee [1, 17]. While these factors may predict a high likelihood of increased patellar tilt and maltracking in kinematically aligned knees, we hypothesise that in accordance with improved functional outcomes and pain scores, there will be fewer patellar tracking issues and fewer required lateral releases. The aim of this study is to compare patellar tracking following kinematic and mechanical TKA via comparison of incidence of lateral joint line release and patellar release, as well as patellar tilt angle and displacement on the sunrise X‐ray view.

## METHODS

A retrospective chart review was undertaken to include patients from one high‐volume tertiary academic medical centre to investigate the incidence of patellar maltracking following mechanical versus kinematic TKA. The study protocol was approved by the governing institutional review board (IRB). Two hundred and fifty‐two patients were identified who underwent primary TKA between 2017 and 2022 by a single fellowship‐trained arthroplasty surgeon at a single institution. Thirty patients were excluded due to lack of necessary X‐rays or TKA following trauma. One hundred and three operations were performed via mechanical alignment and 119 were performed with kinematic alignment. The surgical technique was an unrestricted calipered verified manual technique [14].

**Figure 1:**
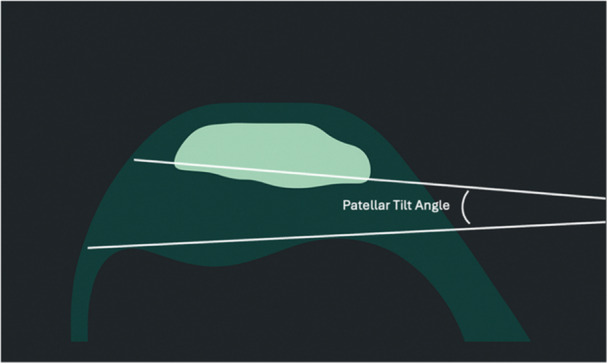
Measurement of patellar tilt angle on sunrise X‐ray view.

**Figure 2:**
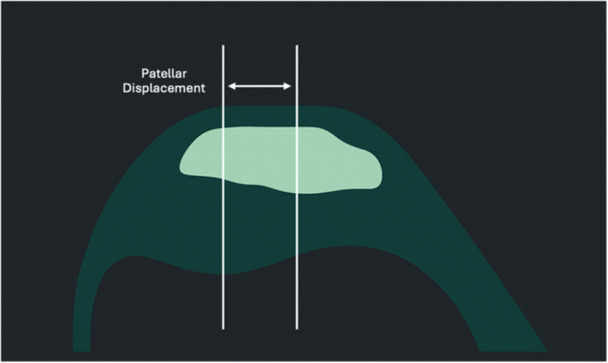
Measurement of patellar displacement on the sunrise X‐ray view.

Retrospective chart review was conducted via the electronic medical record. Data collected included non‐identifiable demographic data including age, gender, sex and race. Surgical data collected included the type of surgical method performed, radiographic imaging angles and incidence of lateral release during operation. Of the 222 patients who underwent surgery, 119 patients had KA‐TKA and 103 patients MA‐TKA (Table 1). Patients were mostly female (58.8%), with an average age of 66 years old at the time of surgery. The mean body mass index (BMI) was 33.8 kg/m^2^, with 15.1% of patients having a BMI greater than 40. Radiographic measurements were obtained from short leg radiographs given full length radiographs were not obtained. Measurements collected included the mechanical distal femoral angle (dFA), proximal tibial angle (pTA), patellar tilt angle and patellar displacement pre‐ and post‐operatively [1]. Femoral and tibial angles were measured from the X‐ray anterior‐posterior view, while the patellar tilt angle and displacement were measured from the sunrise view (Figures 1 and 2). The preoperative angles were taken from the images most proximal to index surgery data, and the postoperative angles were measured from the X‐rays 6 weeks following surgery, if available. If unavailable, measurements would be collected from the most recent post‐operative imaging and accounted for in the statistical analysis. Varus or valgus arthritic patterns were determined based on wear pattern and alignment on short leg radiographs. The respective angles and patellar displacement were measured by two investigators, with patients being randomly divided between the two reviewers. Inter‐rater agreement was confirmed via verification of 20% of images.

**Table 1:** Patient demographics and pre‐operative alignment measurements.

	Kinematic alignment (*n* = 119)	Mechanical alignment (*n* = 103)	*p*‐value^a^
Sex			
Male (%)	49 (41.2)	35 (34.0)	
Female (%)	70 (58.8)	68 (66.0)	
Body mass index			
<40 (%)	101 (84.9)	88 (85.4)	
>40 (%)	18 (15.1)	15 (14.6)	
Mean (SD)	33.8 (5.8)	33.3 (6.0)	0.86
Anatomic alignment			
Valgus (%)	23 (19.3)	25 (24.2)	
Varus (%)	96 (80.7)	78 (75.7)	
Pre‐operative measurements			
Mean distal femoral angle (SD)	82.3 (3.2)	82.6 (2.9)	0.86
Mean tibial angle (SD)	84.6 (3.5)	84.9 (4.17)	0.17
Mean patellar tilt (SD)	4.4 (3.4)	3.7 (3.0)	0.08
Mean patellar displacement (SD)	4.1 (2.3)	4.0 (2.9)	0.34

*Note*: Statistical significance is defined as *p* < 0.05.

Abbreviation: SD, standard deviation.

^a^ From the Wilcoxon rank sum test.

Incidence of lateral patellar release as well as lateral joint line soft tissue release were recorded from the operative reports. While the lateral joint line soft tissue releases were not specifically performed for patellar tracking, it was included in this study as the authors feel this may improve patellar tracking. Patellar resurfacing was also recorded. The decision to resurface the patella was determined by surgeon discretion guided by cartilage wear, bony anatomy, patient preference and pre‐operative pain location.

### Statistical analysis

Baseline characteristics and pre‐operative measurements were stratified by alignment group (kinematic alignment [KA] or mechanical alignment [MA]) and compared using chi‐square tests for categorical variables (sex, BMI category, anatomic alignment and resurfaced status) and Wilcoxon rank‐sum tests for continuous variables (BMI, pre‐operative dFA, pTA, patellar tilt and patellar displacement). Post‐operative measurements (dFA, pTA, patellar tilt, patellar displacement and changes in patellar displacement and tilt) were similarly stratified and compared using Wilcoxon rank‐sum tests. The incidence of intraoperative procedures (patellar release, lateral joint line release and patellar resurfacing) was compared between groups using chi‐square tests.

To evaluate the adjusted effects of alignment on patellar tracking outcomes, generalised linear models with a gamma distribution and log link function were used to account for the skewed, non‐normal distribution of the outcomes, as determined by exploratory analysis. Due to the presence of negative values in the outcome variables, a constant (14.52) was added to ensure all values were positive, as required for the gamma distribution. Separate models were fitted for the change in patellar displacement and the change in patellar tilt as the dependent variables. The models included the following predictors: alignment technique (KA or MA, with MA as the reference), sex (female or male, with male as the reference), BMI (<40 or ≥40 kg/m^2^, with ≥40 as the reference), patellar resurfacing (yes or no, with no as the reference) and patellar release (yes or no, with no as the reference). A sensitivity analysis was performed by including post‐surgical dFA as a covariate to assess its impact on the effect of alignment; this did not alter the findings (rate ratio for alignment on patellar tilt: 0.97 without dFA vs. 0.99 with dFA; rate ratio for alignment on patellar displacement: 0.99 without dFA vs. 0.99 with dFA). Rate ratios, 95% confidence intervals (CIs) and *p*‐values were calculated for each predictor using the Wald chi‐square test. Statistical significance was defined as *p* < 0.05. All analyses were performed using SAS (version 9.4, SAS Institute).

## RESULTS

Two hundred and twenty‐two patients were analysed, with operative dates between January 2017 and 2023 by a single arthroplasty surgeon. The majority of knees were in varus alignment prior to surgery (*n* = 174, 78.3%). Of those who underwent kinematic TKA, 80.7% were in varus pre‐operative alignment, compared to 75.5% of the patients undergoing mechanical TKA. The mean pre‐operative dFA and mean pre‐operative tibial angles for KA knees were 82.3 (standard deviation [SD] 3.2), 84.6 (3.5) and 82.6 (2.9), 84.9 (4.17) for MA knees, respectively. The mean patellar tilt and displacement for KATKA was 4.4 (SD 3.4) and 4.1 (2.3), while MATKA reported values of 3.7 (3.0) and 4.0 (2.9), respectively. There was no statistically significant difference in pre‐operative patellar tilt (*p* = 0.08) or patellar displacement (*p* = 0.34) between kinematic and mechanical knees.

Analysis of intraoperative outcomes showed incidence of patellar retinacular release to be 0.8% (*n* = 1) and 4.9% (*n* = 5) in KA‐TKA and MA‐TKA, respectively (*p* = 0.10) (Table 2). Mechanically aligned knees had a significantly greater frequency of lateral joint line release relative to kinematically aligned knees, with 3.9% of patients requiring release (*p* = 0.04) and 0% in the KA group. Patellar resurfacing was also significantly more common in mechanical alignment. 65.1% of mechanical patients compared to 26.1% of kinematic patients underwent patellar resurfacing (*p* < 0.0001).

**Table 2:** Incidence of intraoperative patellar release, joint line release and patellar resurfacing to assess for intraoperative patellar maltracking.

	Kinematic alignment (*n* = 119)	Mechanical alignment (*n* = 103)	*p*‐value^a^
Patellar release (%)	1 (0.8)	5 (4.9)	0.10
Lateral joint line release (%)	0	4 (3.9)	0.04
Patellar resurfacing (%)	31 (26.1)	67 (65.1)	**<0.0001**

*Note*: Bold indicates statistical significance (*p* < 0.05).

^a^ From chi‐square test.

The mean postoperative dFA and mean postoperative tibial angle for KA‐TKA knees were 82.6 (2.7), 86.6 (2.4) and MA‐TKA knees 85.4 (2.1) and 87.2 (1.8), respectively (Table 3). There was a significantly lower mean postoperative dFA in mechanically aligned knees, resulting in a less valgus angle (*p* < 0.0001). There was no statistically significant difference between postoperative mean tibial angles. The mean patellar tilt after TKA was 2.7 (2.5) for kinematic and 3.2 (4.2) for mechanical knees (*p* = 0.74). Similarly, the mean postoperative patellar displacement was 3.3 (1.9) for kinematic and 3.6 (2.7) for mechanical with no significant difference between the two groups (*p* = 0.23). Further, the difference in patellar tilt and displacement from pre‐ to post‐op was measured. Neither the delta of patellar tilt nor patellar displacement between the two groups was statistically significant (*p* = 0.07, *p* = 0.22).

**Table 3:** Post‐operative measurements of knee alignment and patellar tracking.

	Kinematic alignment (*n* = 119)	Mechanical alignment (*n* = 103)	*p*‐value^a^
Mean distal femoral angle (SD)	82.6 (2.7)	85.4 (2.1)	**<0.0001**
Mean tibial angle (SD)	86.6 (2.4)	87.2 (1.8)	0.09
Mean patellar tilt (SD)	2.7 (2.5)	3.2 (4.1)	0.74
Mean patellar displacement (SD)	3.3 (1.9)	3.6 (2.7)	0.23
Delta patellar tilt	−1.72 (3.3)	−0.40 (4.9)	0.07
Delta patellar displacement	−0.81 (2.8)	−0.48 (2.5)	0.22

*Note*: Bold indicates statistical significance (*p* < 0.05). Delta patellar tilt = (post‐operative patellar tilt—pre‐operative patellar tilt). Delta patellar displacement = (post‐operative patellar displacement—pre‐operative patellar displacement).

Abbreviation: SD, standard deviation.

^a^ From Wilcoxon rank sum test.

### Multivariable analysis of patellar tracking outcomes

The adjusted effects of alignment and other patient and operative characteristics on patellar tracking outcomes are presented (Table 4). For the change in patellar tilt, KA was not significantly different from MA, with a rate ratio of 0.97 (95% CI: 0.92–1.03, *p* = 0.32). This finding remained consistent in a sensitivity analysis controlling for post‐surgical dFA, where the rate ratio for KA versus MA was 0.99 (95% CI: 0.93–1.05, *p* = 0.68). Patellar release was associated with a significant increase in the change in patellar tilt (rate ratio = 1.71, 95% CI: 1.25–2.35, *p* = 0.0009), indicating a 71.12% increase in the expected change in patellar tilt for patients who underwent patellar release compared to those who did not. Other predictors, including sex (rate ratio = 1.00, 95% CI: 0.95–1.05, *p* = 0.91), BMI (<40 vs. ≥40 kg/m^2^; rate ratio = 0.99, 95% CI: 0.92–1.07, *p* = 0.81) and patellar resurfacing (not resurfaced, rate ratio = 1.00, *p* = 0.80) had no statistically significant effect on patellar tilt. No significant difference was found in patellar displacement when controlling for alignment, sex, BMI, patellar resurfacing or patellar release (*p* = 0.80, 0.76, 0.87, 0.60, 0.72, respectively).

**Table 4:** Measurements of patellar tracking controlled for various patient and operative characteristics.

	Rate ratio	Confidence interval (95%)	*p*‐value^a^	*p*‐value** controlling for distal femoral angle
Patellar Tilt				
Alignment (kinematic)	0.97	0.92–1.03	0.32	0.68
Sex (female)	1.00	0.95–1.06	0.91	0.98
Body mass index (BMI) (<40)	0.99	0.92–1.07	0.81	0.78
Patellar resurfacing (Not resurfaced)	0.10	0.94–1.05	0.89	0.80
Patellar release	1.71	1.25–2.35	**0.0009**	**0.0006**
Patellar displacement				
Alignment (kinematic)	0.99	0.94–1.05	0.79	0.80
Sex (female)	1.01	0.96–1.06	0.77	0.76
BMI (<40)	0.99	0.93–1.07	0.86	0.87
Patellar resurfacing (Not resurfaced)	0.98	0.93–1.04	0.59	0.60
Patellar release	1.06	0.77–1.46	0.71	0.72

*Note*: Bold indicates statistical significance (*p* < 0.05).

^a^ From Wald Chi‐square regression adjusted for baseline characteristics. Alignment: kinematic or mechanical; sex: female or male; BMI: >40 or <40; patellar resurfacing: Not resurfaced or resurfaced; Patellar release: Yes or No. p** = controlling for distal femoral angle.

## DISCUSSION

Mechanically aligned TKA has been considered the gold standard technique for TKA for decades [26]. Kinematic TKA has emerged as a new operative approach which focuses on restoring a patient's native anatomy [26]. KA‐TKA has been thought to improve ligamentous balance and minimise the need for soft tissue release as the components will align more closely with each individual patient's anatomy. However, many of the reported complications of KA are related to patellofemoral tracking [2]. This study investigated patellar tracking in mechanical versus kinematic TKA. We found a greater incidence of lateral joint line releases in those undergoing mechanical relative to kinematic TKA. Further, no significant difference occurred in patellar tilt nor displacement between the two techniques.

This data shows that while releases may be indicated more often for mechanically aligned knees, overall patellar tracking is comparable between the two methods. These findings are consistent with a study by Sun et al., which analysed 234 patients who underwent KA‐TKA or MA‐TKA [31]. They found that the patellar lateral retinacular release ratio was greater in the MA (25.4%) relative to KA (6.7%) group without any difference in radiologic parameters of patellar maltracking. Multiple other studies have compared patella‐femoral tracking between KA and MA knees, including a study by McEwen et al. who found that patients receiving MA‐TKA were significantly more likely to require a release compared to KA‐TKA patients (*p* = 0.02) [22]. Further, a similar study of 200 TKAs found that patients who underwent KA‐TKA had significantly fewer soft tissue releases compared to MA‐TKAs (10% vs. 49.2%, *p* < 0.0001) [2]. Finally, a study by Fernadez et al. compared Insall‐Salvati and Blackburne‐peel ratios between kinematic and mechanical knees pre‐ and post‐operatively to assess sagittal patella‐femoral knee alignment, and concluded that both ratios were more accurately reproduced with kinematic knees, suggesting improved patella‐femoral tracking [12]. These studies all reported different outcome measures to account for patellar maltracking. Our study was unique and offers new and valuable information in that it assessed patellar tilt and displacement, two variables that were not accounted for in previous studies.

These results are somewhat counterintuitive because a kinematically aligned knee internally rotates the femoral component, internally rotates the tibial component, increases the q‐angle and aims the trochlear more medial as the valgus alignment increases. However, mechanical knees tend to under‐resect the lateral distal femoral condyle, which places stress on the lateral tissue and may tilt the patella as the knee comes into flexion. The relative effect of these variables likely depends on the specific patient's native anatomy, knee technique, trochlear design, among other variables, which is likely why there is no major difference in patellar tilt between the cohorts. Anecdotally, it does seem that kinematically aligned knees tend to have patellar instability early in flexion due to a more medialized trochlea which never adequately captures the patella. While mechanically aligned knees have issues later in the flexion arc as under resection of the lateral distal femoral condyle stresses the lateral tissues and the patella is ‘pulled’ laterally out of the groove.

However, multiple factors related to implant design and technique also play a role in patellar tracking. Factors that may impact the tracking include the trochlear angle, component rotation, femoral component flexion, femoral component mediolateral position, the patellar component design and the trochlear groove depth and position on an implant, amongst many other factors [23]. A study by Schindler et al. demonstrated that components with a shallow trochlea groove may increase the risk of maltracking [29]. Similarly, a study of 100 native knee joints was analysed to determine how lateral dFA relates to patellar tilt and lateral trochlear inclination [27]. They found that the dFA did not correlate with the patellar tilt while increasing posterior condyle angles resulted in a flatter trochlear inclination. Finally, even the patellar components can have an impact on patellar tracking. A multivariate analysis of 878 primary TKAs showed that a greater patellar component size was associated with 37% decreased risk of patellar tilt [16]. Further, they found that greater external rotation of the femoral component significantly decreased the patellar tilt. While kinematic versus mechanical technique may have an impact on patellar tracking and displacement, it is essential to approach patellar alignment from multiple anatomical, biomechanical and surgical perspectives to ensure optimal implant alignment and functional outcomes.

There are several limitations to consider in this study, primarily related to its retrospective design. The patients in the mechanically aligned and kinematically aligned groups were retrospectively analysed, and the individual postoperative measurements obtained for each subject were collected in a non‐blinded manner. Therefore, to better compare patellar tracking and tilt following KA‐TKA and MA‐TKA, subjects should be evaluated in the setting of a randomised control study. Similarly, lateral patellar release measurements and information regarding the efficacy of the patellar tracking was collected during the surgical procedure in the operating room, which can be subjective. Without an objective method to assess these variables, there may be minor discrepancies correlating the patellar tracking with MA versus KA. Furthermore, the patients assessed were all affiliated one high‐volume institution where they received treatment. Similarly, both MA‐TKA and KA‐TKA patients in this study were operated on by a single orthopaedic fellowship‐trained surgeon, and different physicians may have different surgical approaches or techniques for these procedures. Surgeon discretion was used in determining patellar resurfacing rather than a set criterion which may introduce further bias. Thus, these results may not be generalisable to a larger population. Finally, radiographs used in analysis were short leg films as full length radiographs were not available. Future studies should be conducted to analyse if patients develop patellar maltracking after initial post‐op visits, as well as analyze the survivorship free of revision. Finally, patients were selectively resurfaced at the discretion of the surgeon which may have also influenced the results.

## CONCLUSION

Patellar tracking was found to be comparable between mechanical and kinematic TKA, with no significant difference in incidence of patellar release, patellar tilt nor patellar displacement. Mechanically aligned TKA required a non‐statistically significant increased incidence of lateral joint line release. Overall, these results support that MA‐TKA and KA‐TKA both result in satisfactory patellar tracking with possible reduced need for patellar and lateral joint line releases in KA‐TKA.

## AUTHOR CONTRIBUTIONS

**Meredith Benson**: Data collection; manuscript curation; project oversight. **Chase Gornbein**: Data collection; manuscript curation. **Mitchell Pfennig**: Data collection; manuscript curation; project oversight. **Steven Denyer**: Biostatistics; manuscript curation; project oversight. **Nicholas Brown**: Project oversight; manuscript review.

## FUNDING INFORMATION

The authors have no funding to report.

## CONFLICT OF INTEREST STATEMENT

Nicholas Brown: Royalties: Microport, Link, Corin. Paid consultant: Depuy, Corin, Link, Microport. The remaining authors declare no conflicts of interest.

## ETHICS STATEMENT

This project was approved by the institutional review board (LU# 217467).

## Data Availability

The data that support the findings of this study are available on request from the corresponding author. The data are not publicly available due to privacy or ethical restrictions.
